# Influence of Braking Speed on the Friction and Wear Characteristics of High-Speed Railway Braking Materials under High Ambient Humidity Conditions

**DOI:** 10.3390/ma16176026

**Published:** 2023-09-01

**Authors:** Lei Ma, Meixian Zhang, Siyuan Ding, Yiding Ou

**Affiliations:** Key Laboratory of Fluid and Power, Machinery of Education, School of Mechanical Engineering, Xihua University, Chengdu 620039, China; zhangmeixian2023@163.com (M.Z.); dsy20221105@163.com (S.D.); 17361009915@163.com (Y.O.)

**Keywords:** high-speed rail braking materials, friction and wear characteristics, high ambient humidity, braking speed, disc surface temperature

## Abstract

The friction and wear tests of high-speed railway braking materials for a variety of braking speeds (600, 400, and 200 rad/min) at 65% and 98% RH RH (RH: relative humidity) were carried out utilizing a friction-testing machine and humidity generator. The research results indicate that braking speeds and ambient humidity have a prominent influence on the friction and wear characteristics of high-speed railway braking materials. At 65% and 98% RH, the lower the braking speed, the lower the wear rate, and the better the wear resistance property of the braking material. Furthermore, at 600 rad/min, the wear rate of the braking material at 98% RH was smaller than that at 65% RH. However, at 200 rad/min, the wear rate of the braking material at 98% RH was greater compared to that at 65% RH. Concretely, at 600 rad/min, compared with 65% RH, the wear rate to the brake disc at 98% RH was reduced by about 9%, and the brake pin decreased by about 6%. However, at 200 rad/min, compared to 65% RH, the wear rate to the brake disc at 98% RH increased by about 39%, and the brake pin increased by about 37%.

## 1. Introduction

With the advancement in science and technology, the running speed of high-speed railways is constantly increasing, which requires the braking system of high-speed railways to have good braking performance [1]. As the most momentous components of the braking system, the brakes play a critical role in the braking process [2,3]. Numerous studies have found that the wear properties of high-speed rail brake materials are affected by the braking speed, test force, and materials properties [4,5,6,7]. In addition, in the actual service process, high-speed railways are faced with a variety of extreme ambiental challenges, among which ambient humidity is a significant factor [8,9,10]. For instance, ambient humidity in coastal areas is as high as 96% RH [11]. Under high humidity conditions, the wear properties of braking materials usually change, which can have a certain impact on the stability and reliability of the brake performance of high-speed railways. To study the effects of braking speed and ambient humidity on the friction and wear characteristics of high-speed railway braking materials, experts and scholars have conducted extensive investigations [12,13].

Zhang Meixian et al. [14] discovered that at low ambient temperatures, the wear damage to the braking materials of high-speed rails was induced by an increase in the braking speed. Moreover, Peng Tao et al. [15] studied the brake performance of high-speed railway brake pads at different initial braking speeds and found that, with an increase in the braking speed, the wear damage to the brake pad increased. Zhang Chao et al. indicated that with an increase in the braking speed, the brake disc surface temperature increased [16]. In addition, Wang Zhizhong et al. [17] pointed out that the cracks of high-speed rail brake discs are greatly affected by the braking speed. Furthermore, Cheng Dadan et al. [18] used a pin-disc friction testing machine to simulate the braking materials in a dry-friction medium, a water medium, and a snow medium to test the friction coefficient of the braking materials in three different ambients. The results show that the friction coefficients of braking materials under the action of water and snow decreased compared to that of dry friction. Lyu, Yezhe et al. investigated the effects of humidity on the friction behavior of railway braking block materials and proposed that the friction coefficient of brake block materials reduces with a rise in humidity [19]. Furthermore, Djafri M. [20]’s team studied the impact of humidity on brake disc materials and proved that a water film could form on the brake disc surface at a high humidity level. Wang Fang et al. [21] explored the effect of water on brake shoe materials and proposed that at a low braking speed, the friction coefficient can be reduced by 30% compared with dry conditions, but as the braking speed increases, the friction coefficient increases. Meanwhile, it has been indicated by Le, Wan Kyu et al. [22] that the friction coefficients of braking materials could be affected under humid air conditions.

At present, for the friction and wear characteristics of high-speed rail braking materials under extreme conditions, researchers have mainly concentrated on high-temperature and low-temperature conditions, and there are also few studies under high humidity conditions [23]. In addition, most researchers study high-speed railway braking materials from a single perspective (braking factors or ambient conditions), and there are still fewer that consider the coupling of the two. Therefore, it is of great significance to investigate the effects of the braking speed on the friction characteristics of high-speed rail braking materials under high humidity conditions.

The aim of this paper is to study the effects of a variety of braking speeds on the friction and wear characteristics of high-speed rail brake materials in a range of humidity conditions. A friction-testing machine and humidity generator were utilized to carry out the tests on brake materials at speeds of 600, 400, and 200 rad/min and at 65% RH and 98% RH RH. Meanwhile, the wear characteristics of brake materials under a variety of braking speeds and ambient humidities were investigated, including the friction coefficient, wear rate, wear morphology, and disc sample surface temperature. The research results can provide a reference for high-speed railways to select braking speeds in high ambient humidity and offer theoretical support and basic guidance for high-speed railways to safely operate under high ambient humidity.

## 2. Test

### 2.1. Braking Materials

**Figure 1:**
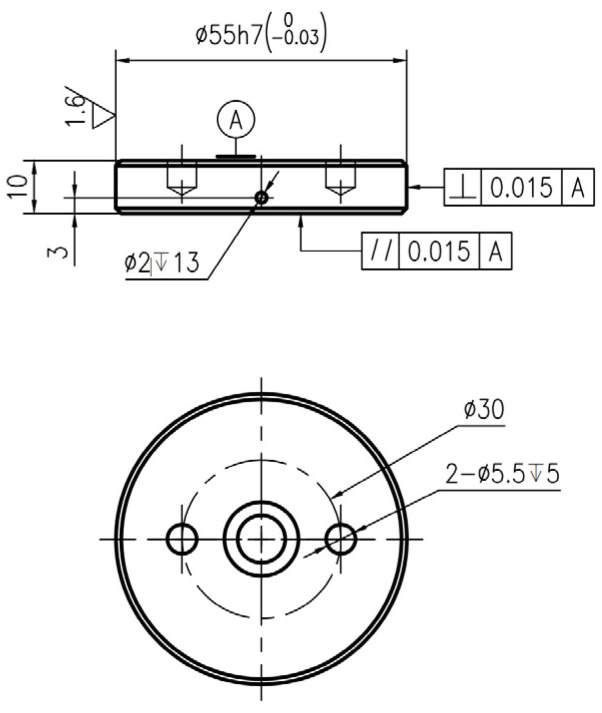
Structure diagram of the brake disc sample (unit: mm).

**Figure 2:**
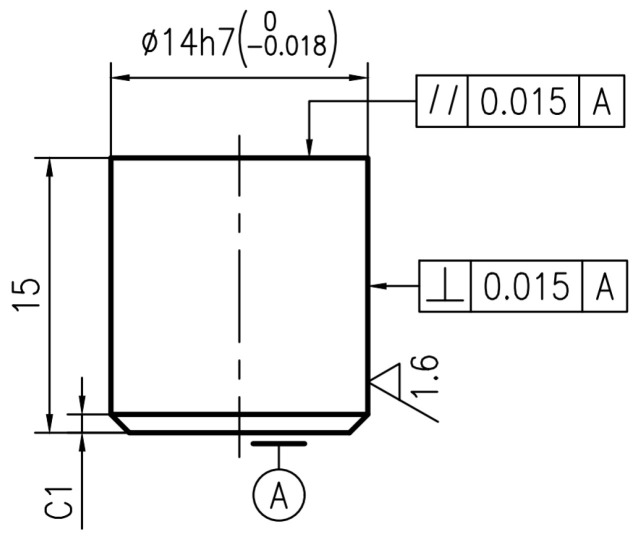
The external dimensions of the brake pin sample (unit: mm).

The test materials for the disc sample and pin sample were obtained from a high-speed railway (CRH380) in China. The disc materials are forged steel. The pin materials are copper-based powder metallurgy. The main chemical compositions of the disc sample and pin sample are displayed in Table 1. Furthermore, Figure 1 and Figure 2 indicate the structure diagrams of the disc sample and the pin sample. Specifically, the brake disc materials are designed in a disc shape with a diameter of 55 mm and a thickness of 10 mm. In addition, the side of the disc has a hole with a diameter of 2 mm and a depth of 13 mm to place the temperature sensor. There are two holes with a diameter of 5.5 mm and a depth of 5 mm at the bottom of the disc sample, which are used to fix the disc sample on the fixture. The pin materials are machined into a cylindrical shape with a diameter of 14 mm and a thickness of 15 mm.

### 2.2. Test Equipment

**Figure 3:**
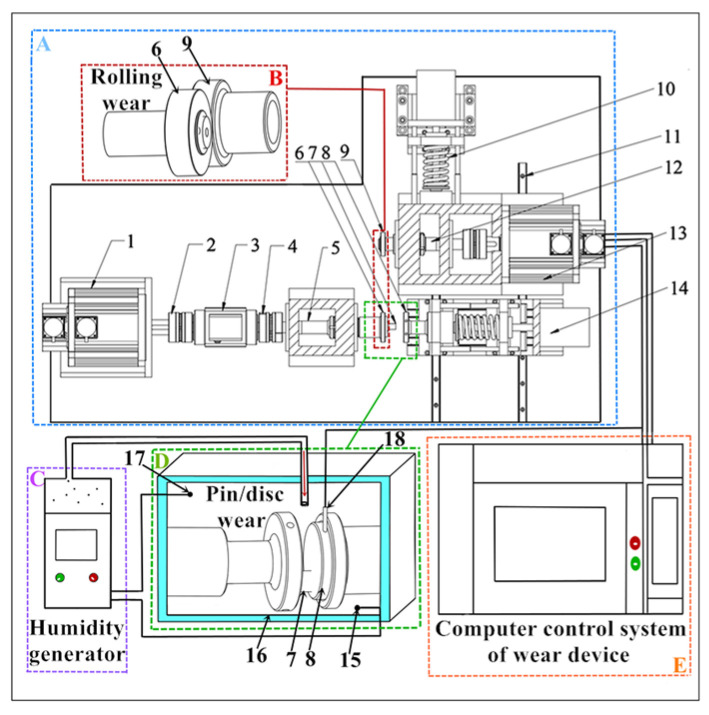
The schematic diagram of friction testing equipment. (A): Friction test machine; (B): Rolling samples seal chamber; (C): Humidity generator; (D): Pin/disc samples seal chamber; (E): Computer control system; (1): Servo motorI; (2): Couplings; (3): Torque sensor; (4): Force sensor; (5): Drive shaft; (6): Rolling sampleI; (7): Pin sample; (8): Disc sample; (9): Rolling sampleII; (10): Driving screw; (11): Sliding rail; (12): Rotation shaft; (13): Servo motorII; (14): Servo motorIII; (15): Bottom humidity sensor; (16): Insulated cotton; (17): Top humidity sensor; (18): Temperature sensor.

Figure 3 is the schematic diagram of the friction testing equipment used in this paper. The friction testing equipment includes a multifunctional friction test machine (A) (Shandong Province, China) and a humidity generator (C). Among them, (A) includes three parts: (B), (D) and (E). During the test, (1) drives (2) rotates, and the torque is monitored by (3). At the same time, the test force is monitored by (4). And (5) transmits motion and force to (6) and (7). In addition, (8) and (9) are located to the right of (A). They change position at (10) and (11) and transmit force and motion through (12) and (13). And the function of (14) is to move the specimen and bring it into contact to perform the wear test. Additionally, (15) is a sensor of (C) that monitors the humidity at the bottom of (D), while (16) is used to prevent heat loss. Moreover, (17) is another sensor of (C), which is used to monitor humidity at the top of (D). And (18) is a sensor used to measure the temperature value on the disc surface. Meanwhile, the braking speeds, test force, loading speeds, and other parameters required by the test are set using (E). At the same time, the test data in the testing process are fed back to the screen of (E) in real-time. And after reaching the test time, (C) should be turned off first. Then, the test force should be unloaded through (E). When the brake disc sample and brake pin sample are removed, the power supply should be turned off.

### 2.3. Test Process and Details

In order to study the influence of a variety of braking speeds on the friction properties of the braking materials in a range of humidity conditions, in this paper, the braking speeds were set to 600, 400, and 200 rad/min, respectively. Meanwhile, the humidity generator was utilized to maintain an ambient humidity at 98% RH (an error of approximately ±2%). In addition, the friction tests of the braking materials for a variety of braking speeds (600, 400, and 200 rad/min) at 65% RH (natural ambient humidity in Chengdu, Sichuan Province, China) were carried out as the control group. Moreover, the simulation tests of braking materials at low sliding speeds and a low test force do not lead to changes in the wear mechanisms, according to existing research [19]. Therefore, in this test, the loading speed was set at 0.5 m/s, and the test force was 100 N. Table 2 shows the testing parameters.

First, the brake pin sample and brake disc sample were soaked in alcohol and washed in an ultrasonic cleaning machine. After cleaning, a hair dryer was utilized to dry the residual alcohol on the brake disc and brake pin surface. Additionally, the brake disc sample and brake pin sample were placed into a drying cabinet to dry for 8 h. After 6–8 h, the brake disc sample and brake pin sample were weighed via a balance (JA4103, accuracy 0.001 g, China). Additionally, each sample was weighed 6 times and recorded. The average values were taken as the quality of the pin sample and disc sample. Additionally, because the wear amount of the braking materials was small, the ratio of the wear mass to total revolutions was used to represent the wear rate. In addition, the Ultra Depth of Field Optical Microscope (RS-V1, Shanghai, China) was utilized to observe the wear marks on the brake disc sample and brake pin sample.

## 3. Test Results of Braking Materials

### 3.1. Friction Coefficient Analysis

**Figure 4:**
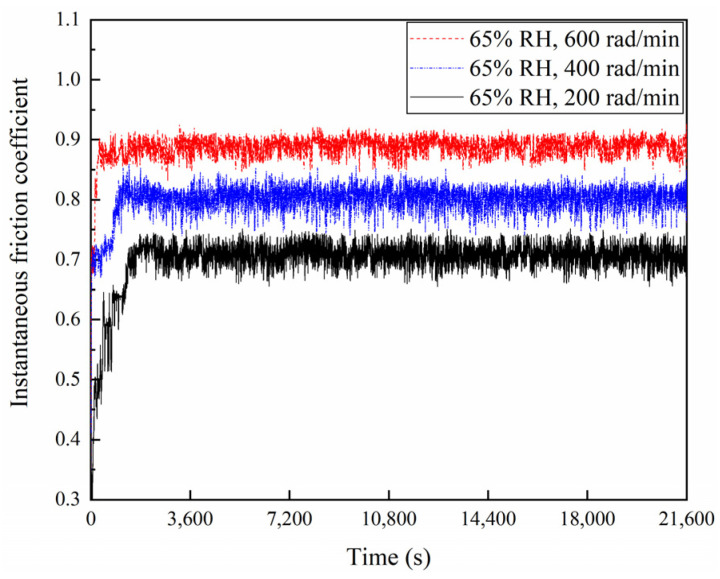
Instantaneous friction coefficients diagram for a variety of braking speeds at 65% RH.

**Figure 5:**
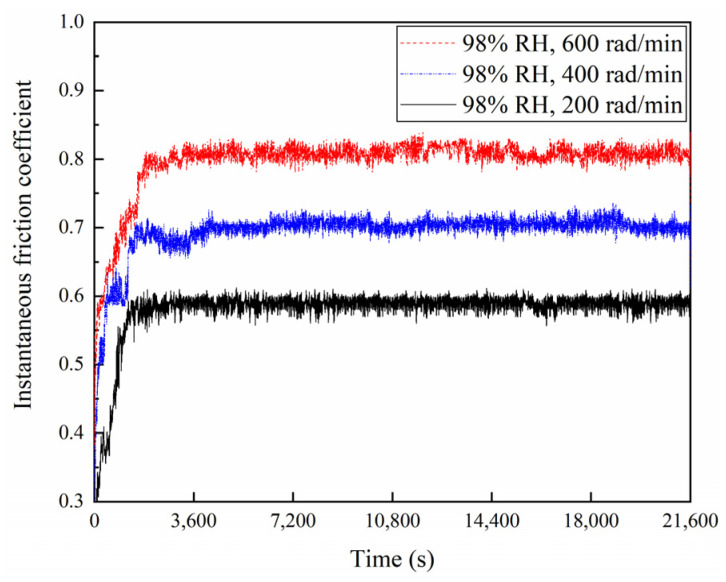
Instantaneous friction coefficients for a variety of braking speeds under 98% RH conditions.

**Figure 6:**
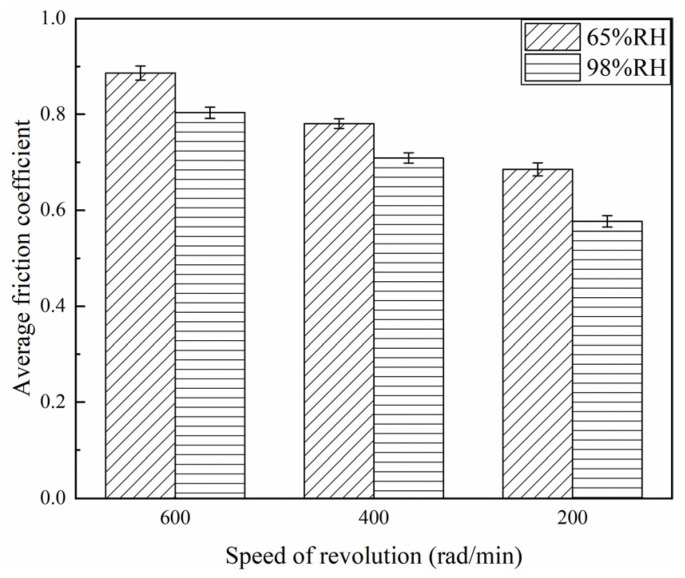
Average friction coefficients for a variety of braking speeds at 65% RH and 98% RH.

The friction coefficients of the braking materials for a variety of braking speeds at 65% RH and 98% RH are displayed in Figure 4, Figure 5 and Figure 6. At 65% RH, the friction coefficients of the braking materials for 600, 400, and 200 rad/min all displayed a rapid increase in the first 1800 s. But it rose the fastest at 600 rad/min, where the slope of the curve is greatest. After 3600 s, these instantaneous friction coefficients gradually became stable and exhibited a slow change. The instantaneous friction coefficient at 65% RH, 600 rad/min, was the largest, followed by 65% RH, 400 rad/min, and the smallest was at 65% RH and 200 rad/min. Moreover, at 98% RH, the friction coefficients increased quickly during the first 1800 s. The friction coefficients showed a slow increase at 600 and 200 rad/min after 1800 s. It displayed a decrease at 400 rad/min. After 3600 s, these instantaneous friction coefficients showed a slow change and gradually became stable. However, the instantaneous friction coefficient was the largest at 98% RH, 600 rad/min, and the smallest at 98% RH, 200 rad/min. In addition, Figure 6 shows the average friction coefficients of the braking material in a range of humidity conditions and a variety of braking speeds. At 65% RH and 98% RH, as the braking speeds decreased from 600 to 400 and then to 200 rad/min, the average friction coefficients diminished successively. At 65% RH, the average friction coefficient at 600 rad/min was 0.89; at 400 rad/min it was 0.78; at 200 rad/min, it was 0.69. But under 98% RH conditions at 600 rad/min it was 0.8; at 400 rad/min, it was 0.71; and at 200 rad/min, it was 0.58.

### 3.2. Wear Rate

**Figure 7:**
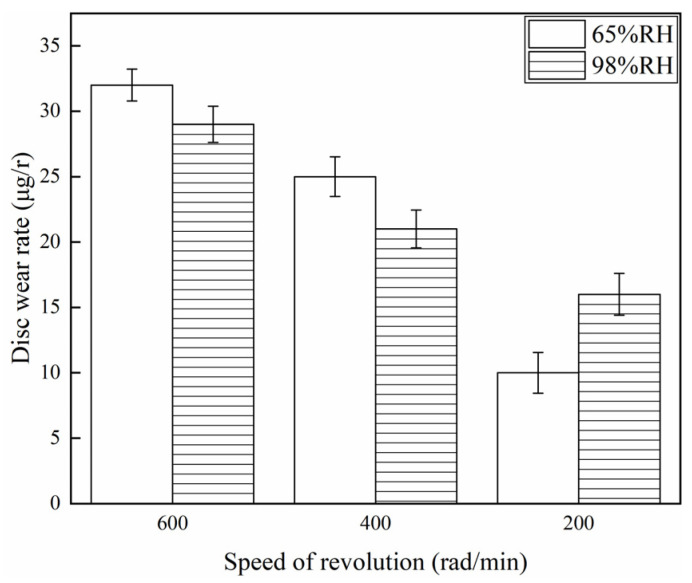
Wear rate to the brake disc for a variety of braking speeds at 65% RH, 98% RH.

**Figure 8:**
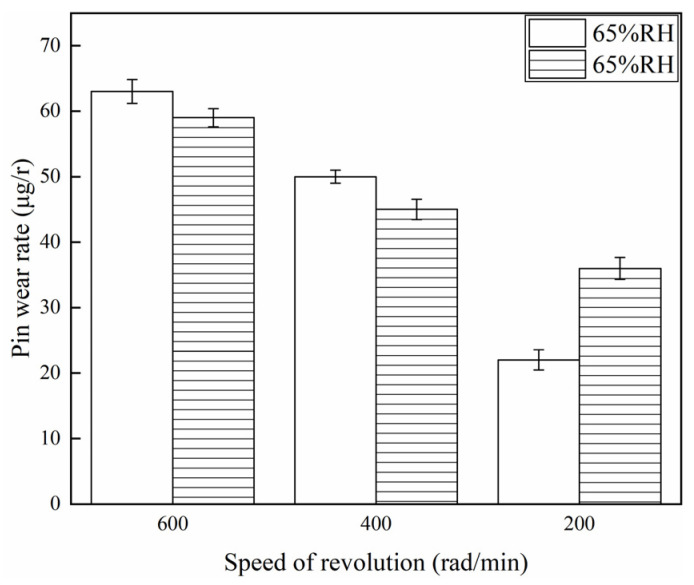
Wear rate to the brake pin for a variety of braking speeds at 65% RH, 98% RH.

Figure 7 and Figure 8 show the wear rate of the brake disc and brake pin for a variety of braking speeds at 65% RH and 98% RH. It is not difficult to find from Figure 7 that when ambient humidities were the same, the wear rate of the disc declined with a reduction in the braking speeds. Under certain braking speed conditions, the wear rate of the brake disc at 98% RH was smaller than that at 65% RH, under 600 and 400 rad/min conditions. But, at 200 rad/min, the wear rate of the brake disc at 98% RH was larger compared to that at 65% RH. Concretely, under 65% RH conditions, the wear rate of the brake disc at 200 rad/min was 10 µg/r, and at 600 rad/min was 32 µg/r. But at 98% RH, the wear rate at 200 rad/min was 16 µg/r, and at 600 rad/min was 29 µg/r. The wear rate of the brake disc was highest at 65% RH, 600 rad/min. And at 65% RH, 200 rad/min was the smallest. Figure 8 shows the wear rate of the brake pin for a variety of braking speeds at 65% RH and 98% RH. The wear rate of the brake pin was much greater than that of the brake disc under the same ambient humidities and braking speed conditions. In addition, under 65% RH conditions, the wear rate of the brake pin at 200 rad/min was 22 µg/r, and at 600 rad/min was 63 µg/r. But at 98% RH, the wear rate at 200 rad/min was 36 µg/r, and at 600 rad/min was 59 µg/r.

### 3.3. Wear Morphology

#### 3.3.1. Wear Morphology to the Brake Disc

**Figure 9:**
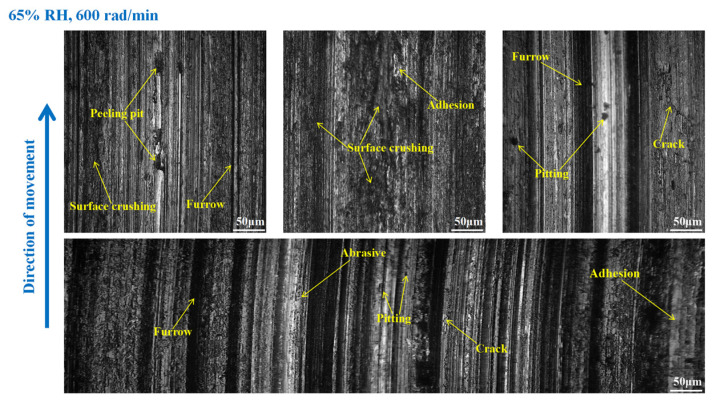
The wear marks to the brake disc at 65% RH, 600 rad/min.

**Figure 10:**
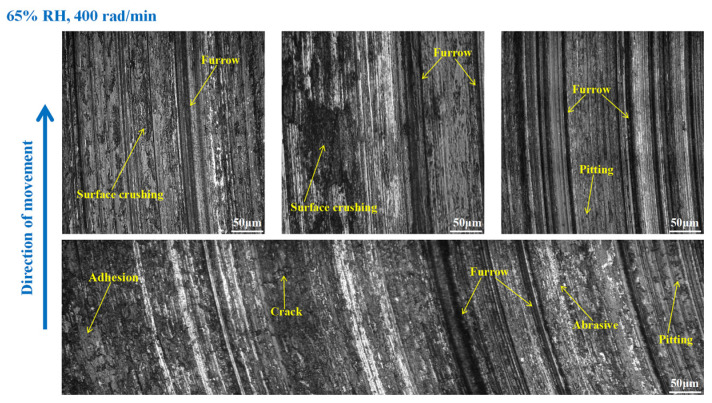
The wear marks to the brake disc at 65% RH, 400 rad/min.

**Figure 11:**
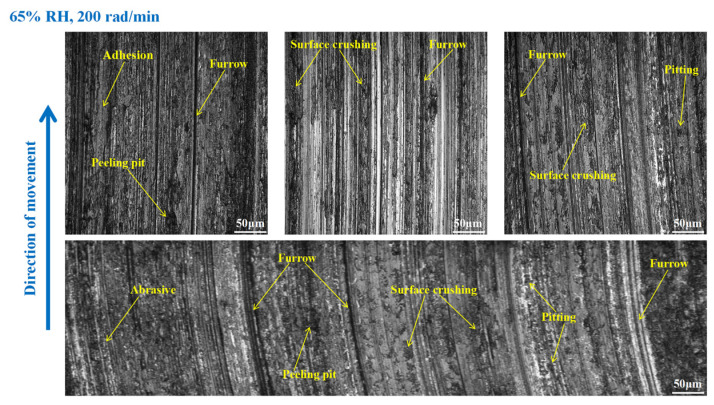
The wear marks to the brake disc at 65% RH, 200 rad/min.

It is obvious from Figure 9 that the wear of the brake disc was serious at 65% RH, 600 rad/min. Pitting, peeling pits, and furrows were identified on the disc surface. In addition, there were cracks, abrasive damage, and adhesion damage around the furrows. At the same time, some heavily worn areas had been crushed. Compared with Figure 9, the surface damage of the brake disc in Figure 10 appears reduced. Specifically, the amount of pitting on the disc surface was diminished, and there were no obvious peeling pits. However, there were still furrows, pitting, capillary cracks, abrasive particles, and adhesion damage on the disc’s surface. In addition, from Figure 11, it is not difficult to find that the surface of the brake disc was smoother than that in Figure 10, and the furrows, pitting, adhesion damage, and abrasive damage were attenuated. The areas of the disc surface that were crushed were reduced.

**Figure 12:**
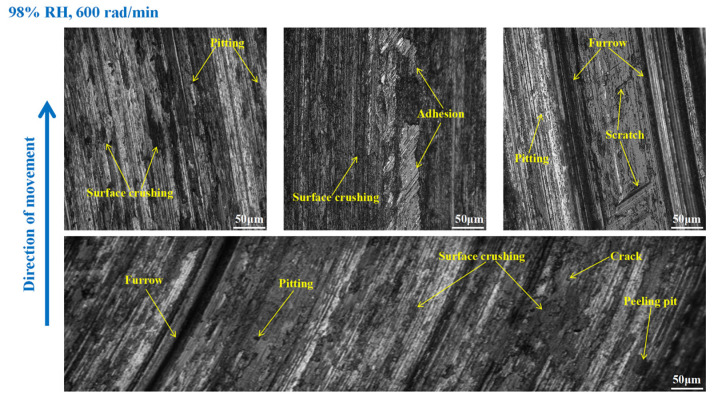
The wear marks to the brake disc at 98% RH, 600 rad/min.

**Figure 13:**
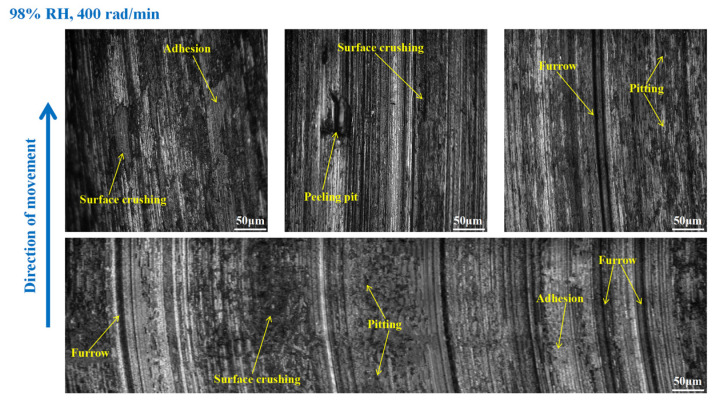
The wear marks to the brake disc at 98% RH, 400 rad/min.

**Figure 14:**
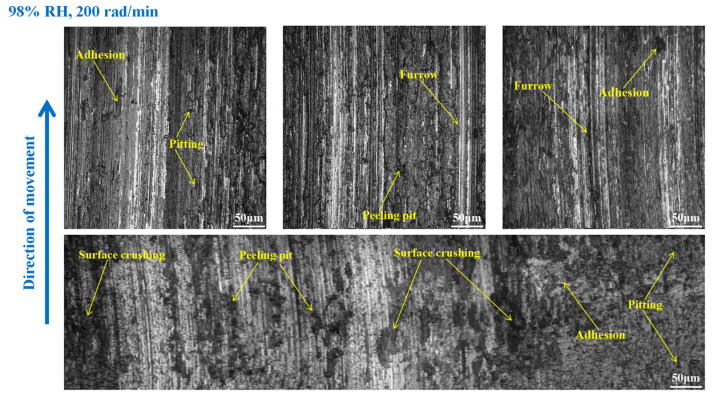
The wear marks to the brake disc at 98% RH, 200 rad/min.

As shown in Figure 12, furrows, pitting, scratches, capillary cracks, peeling pits, adhesion damage, and abrasive damage existed on the disc surface. Meanwhile, some materials fell off from the disc’s surface. However, the wear damage to the disc at 98% RH, 600 rad/min was smaller than that under 65% RH, 600 rad/min, conditions. Compared to Figure 12, the surface damage of the disc in Figure 13 relatively decreased. The wear marks were mainly pitting, furrows, peeling pits, surface crushing, and adhesion damage. Obviously, the wear of the brake disc at 98% RH, 200 rad/min, was small, as shown in Figure 14. The number of furrows, pitting, and peeling pits on the disc surface was reductive. Moreover, the surface crushing and adhesion damage were reduced, and the overall wear was relatively slight.

#### 3.3.2. Wear Morphology to the Brake Pin

**Figure 15:**
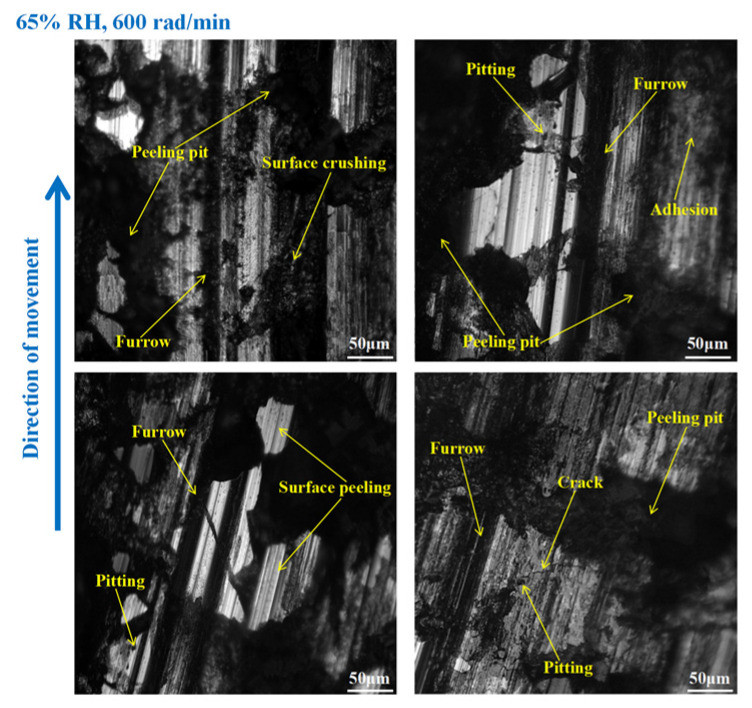
The wear marks of the brake pin at 65% RH, 600 rad/min.

**Figure 16:**
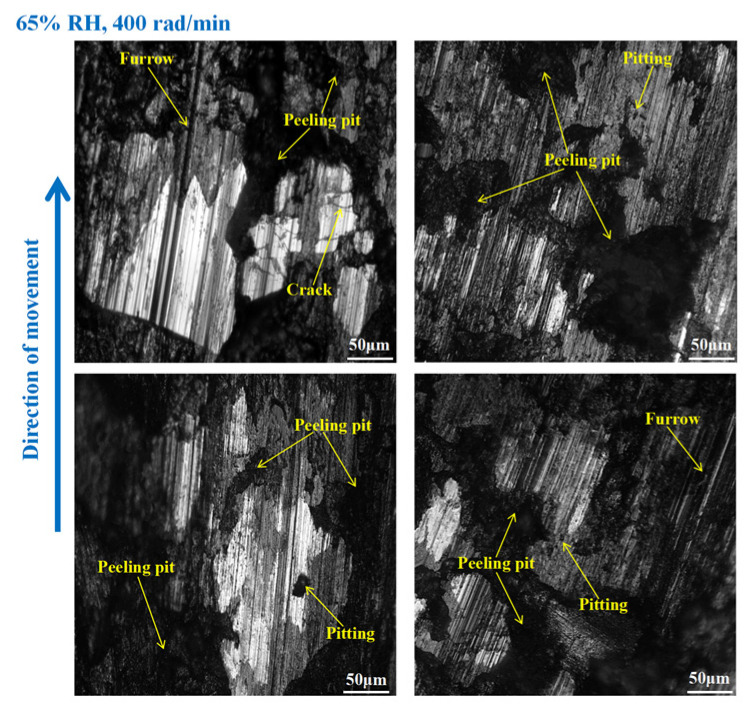
The wear marks of the brake pin at 65% RH, 400 rad/min.

**Figure 17:**
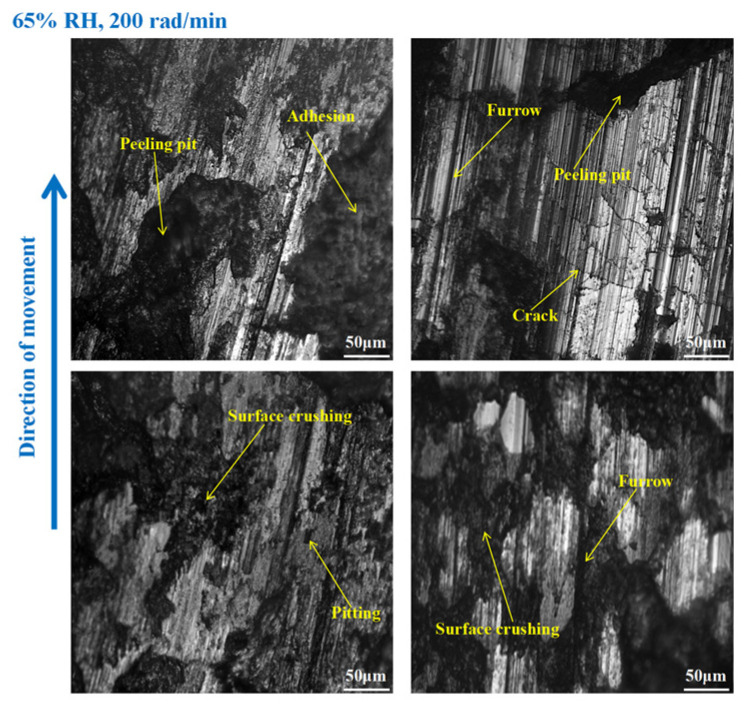
The wear marks of the brake pin at 65% RH, 200 rad/min.

As shown in Figure 15, serious wear damage occurred when the brake pin was at 65% RH, 600 rad/min. There were furrows, cracks, pitting, peeling pits, and adhesion damage to the pin surface. In addition, some areas were crushed, and the materials fell off from the pin surface in clumps, while the friction damage was severe. Compared with Figure 15, in Figure 16, the crushed area of the pin was reduced, and the amount of material falling off from the pin surface decreased. However, the furrows, pitting, cracks, and peeling pits on the surface of the pin were still clearly visible. As displayed in Figure 17, the wear of the pin was significantly diminished compared to that in Figure 16. There is no evident material shedding, and the number of furrows, pitting, and peeling pits is reduced. In addition, the surface crushing and adhesion damage are attenuated.

**Figure 18:**
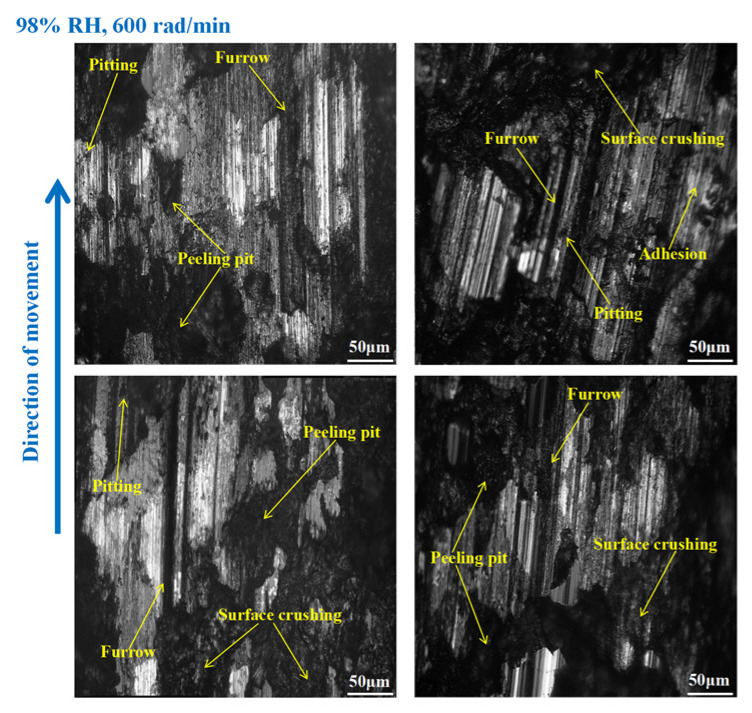
The wear marks of the brake pin at 98% RH, 600 rad/min.

**Figure 19:**
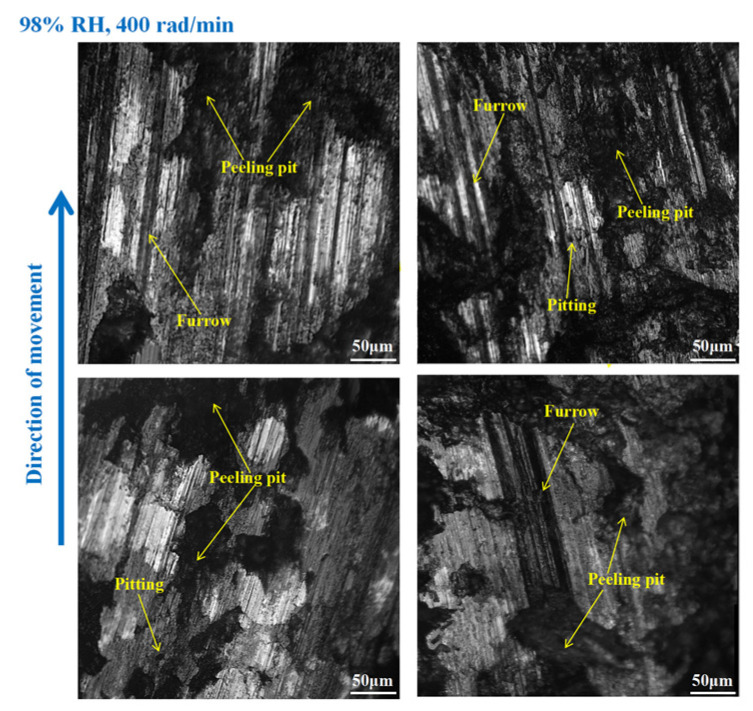
The wear marks of the brake pin at 98% RH, 400 rad/min.

**Figure 20:**
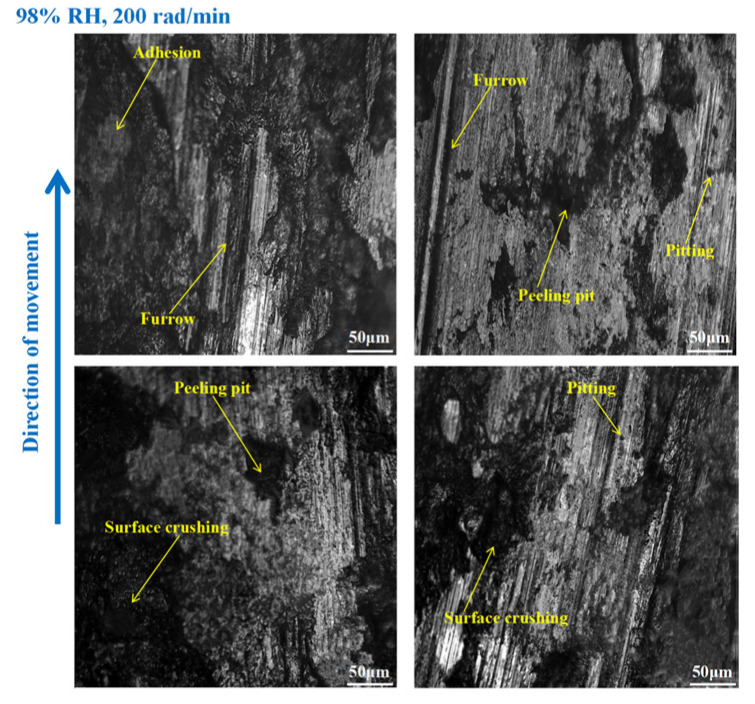
The wear marks of the brake pin at 98% RH, 200 rad/min.

In Figure 18, the pitting and furrows on the pin surface are conspicuous. There are numerous areas that have been crushed, some materials have fallen off from the pin surface, and the peeling pits are evident. However, the wear is lower than that of the brake pin under 65% RH, 600 rad/min conditions. And the same thing is that there is adhesion damage on the brake pin surface. As displayed in Figure 19, the friction damage of the brake pin at 98% RH, 400 rad/min, mainly included pitting, furrows, and peeling pits. Compared to Figure 18, the wear damage to it decreased. Significantly, compared with Figure 15 and Figure 19 above, the overall surface of the brake pin in Figure 20 is relatively flat and less worn. The furrows, pitting, and adhesion damage are attenuated. Meanwhile, the crushing areas and the number of peeling pits are diminished.

### 3.4. Disc Surface Temperature Analysis

**Figure 21:**
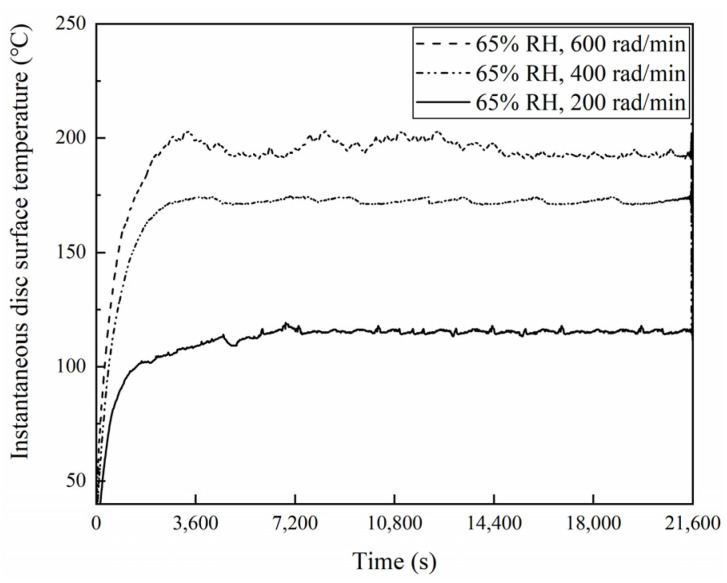
Instantaneous disc surface temperatures for a variety of braking speeds at 65% RH.

**Figure 22:**
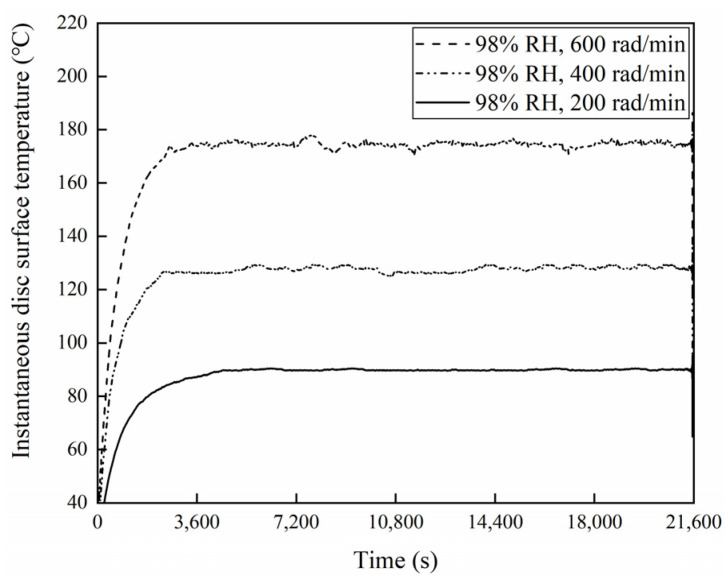
Instantaneous disc surface temperatures for a variety of braking speeds at 98% RH.

**Figure 23:**
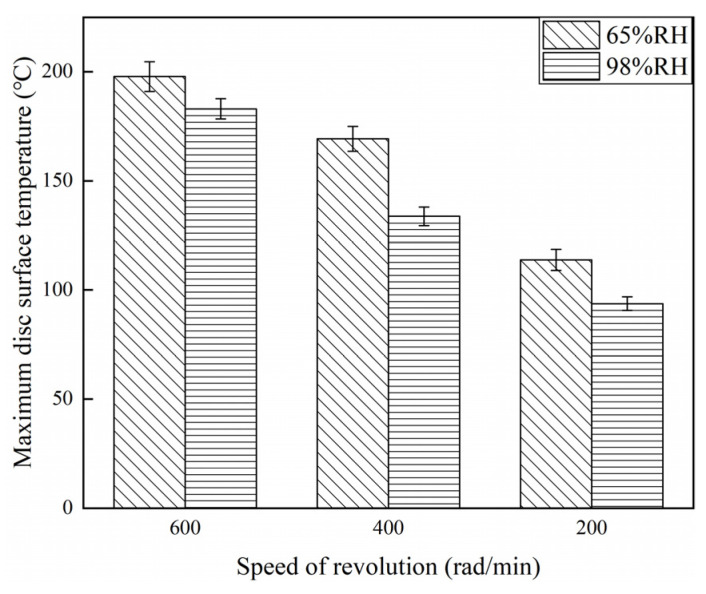
Maximum disc surface temperatures for a variety of braking speeds at 65%, 98% RH.

Figure 21, Figure 22 and Figure 23 exhibit the temperatures on the brake disc surface at a variety of braking speeds in a range of humidity conditions. As shown in Figure 21, when the ambient humidity was 65% RH, the instantaneous disc surface temperatures at 600 rad/min were greater than those at 400 rad/min and larger than those at 200 rad/min. In addition, when the braking speed was 600 rad/min, the disc surface–temperature curve fluctuated greatly before 14,400 s. But at 400 and 200 rad/min, the disc surface temperatures increased first and then became stable. And the temperature curve fluctuation was relatively small. Meanwhile, it is worth noting that as the braking speed was 200 rad/min, the temperatures changed little after 7400 s. Moreover, similar to Figure 21, it is clear from Figure 22 that when the ambient humidity was 98% RH, the disc surface temperatures declined with a reduction in the braking speed. However, significantly, the disc surface–temperature curves fluctuated less at 98% RH compared to that at 65% RH. Especially at 98% RH, 200 rad/min, the brake disc surface temperatures increased first and then became steady. After 7200 s, the disc surface temperatures almost remained unchanged. In addition, compared with Figure 21, it is not difficult to identify that at the same braking speeds, the instantaneous disc surface temperatures were lower at 98% RH than those under 65% RH conditions. Furthermore, Figure 23 displays the maximum disc surface temperatures under a variety of braking speed conditions in a range of humidity conditions. It is evident that at the same ambient humidities, as the braking speed decreased, the maximum disc surface temperatures diminished. In addition, under certain braking speed conditions, the maximum disc surface temperature values were greater at 65% RH compared to that at 98% RH. Among them, under 65% RH conditions, the maximum temperature at 200 rad/min was 113.8 °C, and at 600 rad/min was 197.8 °C. But at 98% RH, the maximum disc surface temperature at 200 rad/min was 93.7 °C, and at 600 rad/min was 182.9 °C.

## 4. Discussions

In this paper, the friction tests of high-speed railway braking materials at 65% and 98% RH under 600, 400, and 200 rad/min conditions were studied utilizing a friction test machine and humidity generator. The results reveal that the braking speed and ambient humidity remarkably affected the friction characteristics of the braking materials.

On the one hand, under certain humidity conditions, it can be observed from Section 3.1 that the friction coefficients of the braking materials reduced with a decrease in the braking speed. The lower the braking speed, the more stable the instantaneous friction coefficient curves of the braking materials were. In addition, at 600 rad/min, the average friction coefficient was the highest, and at 200 rad/min was the lowest. Concretely, at 200 rad/min, compared with 600 rad/min, the average friction coefficients were reduced by about 23% at 65% RH and decreased by about 29% at 98% RH. In addition, Section 3.2 indicates that when the braking speed declined, the wear rate of the brake disc and brake pin both decreased. Under 200 rad/min, and compared with 600 rad/min, the wear rate of the brake disc reduced by about 68% at 65% RH and decreased by about 44% at 98% RH. The brake pin was reduced by about 63% at 65% RH and decreased by about 38% at 98% RH. Meanwhile, in Section 3.3, the wear damage of the braking materials attenuated with a reduction in the braking speed. At 200 rad/min, the wear of the braking materials was slighter than those at 400 and 600 rad/min. Furthermore, in Section 3.4, as the braking speed diminished, the maximum disc surface temperatures decreased. Concretely, at 200 rad/min, compared with 600 rad/min, the maximum disc surface temperatures were reduced by about 42% at 65% RH and decreased by about 49% at 98% RH. Synthesizing the above results under certain ambient humidity conditions and changing the braking speed could influence the friction properties of the braking materials. According to the energy conversion formulas, the braking speed affected the temperature on the brake disc surface [24]. When the braking speed was lower, less heat was generated by friction, which is why the brake disc surface temperature was reduced with a decrease in the braking speed. Moreover, the rise in the braking temperature can change the performance of the braking materials [25]. But the braking materials are less affected under low braking speed conditions.

On the other hand, under certain braking speed conditions, it can be seen from Section 3.1, Section 3.3, and Section 3.4 that the friction coefficients, wear damage, and disc surface temperatures of the braking materials at 98% RH are all smaller than those at 65% RH. This is because, under 98% RH conditions, the humid air condenses and becomes saturated with water droplets. With the rotation of the brake pin sample, these water droplets can adhere TO the surface of the disengaging position [26]. And the water droplets can reduce wear to the braking materials [27]. However, the wear to the braking materials under natural conditions (65% RH) is dry friction, there are no water droplets, and a number of hard particles are exposed on the braking materials’ surface. The presence of these hard particles increases the uneven contact between the brake disc and the brake pin. Therefore, the surface of the braking materials becomes seriously worn, and there are a lot of furrows, pitting, and cracks. In addition, under the action of shearing force, the friction surface is crushing, and some materials fall off from the braking materials surface. There are several peeling pits and adhesion damage. Under high braking speed conditions, the abrasive wear and adhesive wear to the braking materials is aggravated. In addition, under certain braking speed conditions, the wear damage to the braking materials at 98% RH is less than that at 65% RH. Therefore, the number of furrows, pitting, and peeling pits is reductive. Meanwhile, the surface crushing and adhesion damage are reduced. However, at 98% RH, the water droplets adhere to the disc surface. This leads to braking materials becoming more prone to fatigue damage [28]. In conclusion, under 65% RH conditions, the wear mechanisms of the braking materials principally undergo adhesive wear and abrasive wear. And under 98% RH, these conditions principally produce abrasive wear, adhesive wear, and surface fatigue wear.

Considering both braking speed and ambient humidity comprehensively, the wear rate to the braking materials at 98% RH is smaller than that at 65% RH, under 600 and 400 rad/min conditions. But, at 200 rad/min, the wear rate to the braking materials at 98% RH is larger compared to that at 65% RH. Concretely, at 98% RH, compared with 65% RH, the wear rate to the brake disc increases by about 39% and increases by about 37% of the brake pin. The reason for this is that at high braking speeds, the water droplets on the brake disc surface are easily thrown out, and their stay time is short. Meanwhile, as the braking speeds are great, the brake disc surface temperatures are also high. This high temperature can lead to increased wear to the braking materials. However, at 98% RH, 200 rad/min, numerous water droplets adhere to the disc surface. But, due to the slow braking speed, it is difficult to throw them out in a short time, and this exacerbates the surface fatigue damage to the braking materials [29].

## 5. Conclusions

1. Under certain ambient humidity conditions, the friction coefficient, wear rate, wear damage, and the disc surface temperature of the braking materials reduce with a decrease in the braking speed.
2. Under certain braking speed conditions, the friction coefficient to the braking materials at 98% RH is smaller compared to that at 65% RH. However, at 200 rad/min, the wear rate to the braking materials at 98% RH is larger than that at 65% RH.
3. Considering both the braking speed and ambient humidity comprehensively, at a high braking speed, the effect of high humidity on the braking materials is small but significant at low braking speeds.
4. Under 65% RH conditions, at 600, 400, and 200 rad/min, the wear mechanisms to the braking materials principally include adhesive wear and abrasive wear. And under 98%, RH conditions principally involve abrasive wear, adhesive wear, and surface fatigue wear.

According to the above analysis, when running in natural ambient humidity (65% RH) conditions, a high-speed railway should slow down before braking. But in high ambient humidity (98% RH), it is significant to try and avoid braking under low-speed conditions. If it must run in high humidity conditions, high-speed braking should be selected. In addition, in future studies, more speed parameters should be set to find the optimal braking speed under high ambient humidity conditions.

## Figures and Tables

**Table 1:** Chemical elemental composition of the brake disc sample and brake pin sample (wt/%).

Element	Mn	C	Cr	Cu	Si	Ni	O	Fe	S
Brake disc sample	1.02	0.42	0.70	-	0.54	0.08	-	Bal	-
Brake pin sample	-	6.75	3.47	38.85	-	-	20.31	Bal	1.18

**Table 2:** The testing groups and parameters set (repeat 3 times for each group).

Number	Braking Speeds/(rad/min)	Test Humidities/(%)	Test Force/(N)	Loading Speed/(m/s)	Ambient Temperature/(°C)
1	600	65	100	0.5	20
2	400				
3	200				
4	600	98			
5	400				
6	200				

## Data Availability

Not applicable.
